# Advancing Research on Fear of Dementia Through eHealth Solutions in an Aging Society

**DOI:** 10.31083/AP45974

**Published:** 2025-08-22

**Authors:** Huohuo Dai, Jiska J. Aardoom, Niels H. Chavannes, Anke Versluis

**Affiliations:** ^1^Department of Public Health and Primary Care, Leiden University Medical Centre, 2333 ZD Leiden, The Netherlands; ^2^National eHealth Living Lab, 2333 ZD Leiden, The Netherlands

Dementia is a broad category of symptoms arising from changes in brain function. The characteristic features of dementia are difficulties with memory, language, problem-solving, and other cognitive skills, which impair an individual’s ability to carry out routine daily activities [1]. Currently, Alzheimer’s disease and related dementias (ADRD) affect over 55 million people globally, with this number expected to exceed 150 million by 2050 due to population growth and aging [2]. As ADRD becomes more prevalent, public awareness, as well as fear and anxiety surrounding these conditions may also increase.

Fear of dementia is generally identified as the psychological and emotional response to the prospect of developing dementia in the future [3]. Evidence has shown that dementia has surpassed cancer as the most feared condition in the United States [4]. This fear is associated with various negative consequences, such as insomnia [5] and memory failure [6], but may also lead to negative long-term outcomes as elevated levels of fear can cause individuals to delay screening thereby limiting opportunities for early detection and intervention [7]. Despite this, few studies have explored strategies to mitigate this fear, creating an urgent gap in current health research. In an increasingly aging society, it is crucial to address the needs of the older population by identifying effective approaches that enable this growing demographic to benefit from health interventions. It is essential to address the fear of dementia with effective, cost-efficient, and evidence-based interventions, to ensure the efficient use of resources while supporting long-term accessibility and sustainability. By addressing this critical gap, our research aims to contribute to the development of practical solutions, ultimately aiming to advance societal well-being.

eHealth refers to “health services and information delivered or enhanced through the Internet and related technologies” [8], and it’s increasingly utilized for remote, timely, high-quality, and limited-contact care. In our aging society, eHealth interventions hold great promise for improving the health of the older population [9]. However, concerns frequently arise regarding the participation of older adults in eHealth. Many older individuals exhibit lower willingness to participate, possess limited digital skills, and face safety concerns related to comorbidities and polypharmacy [9]. Additionally, researchers do not always effectively reach these populations or employ appropriate recruitment strategies to engage them. Consequently, older adults are often not involved in the development and testing of eHealth tools, which may lead to solutions that do not align with their needs and capabilities, ultimately contributing to the so-called “digital divide” between generations.

Encouragingly, a recent national survey in Canada found that while younger individuals had more knowledge of eHealth, those aged 55 and older were more comfortable using AI for decision support and disease prediction. This indicates a readiness among older adults to engage in eHealth [10]. Additionally, the UN Economic Commission for Europe, along with countries such as the UK and China, has introduced policies—including the “Aging in the Digital Era” policy brief—to improve digital skills among older adults [11]. Moreover, a recent coach-supported mHealth intervention has shown success in reducing dementia risk factors in low- and middle-income countries, as well as among low-income groups in high-income countries, with notable active participation [12].

Despite increasing awareness of the fear of dementia and its associated negative health outcomes among middle-aged and older adults, there is still limited published evidence on effective interventions, particularly eHealth interventions. Encouragingly, the Reducing Fear and Avoidance of Memory Loss (REFRAME) study by Farina *et al*. [7], conducted with participants over 55 years of age in the USA, indicated that eHealth-based psychological interventions can alleviate ADRD-related fears and avoidant coping behaviors in older adults, with benefits extending to broader health outcomes. Additionally, Damico *et al*. [13] implemented a self-guided e-learning Memory and Aging Program for older adults in Canada, which demonstrated good feasibility and acceptability, and resulted in a reduction of age-related memory concerns. However, further research is needed to assess the effectiveness of emerging eHealth interventions in addressing fear of dementia and to evaluate whether their benefits—such as high accessibility, interactivity, effectiveness, and relatively low cost—can be maintained in low- and middle-income countries.

Given the rising prevalence of ADRD and its related anxiety in this rapid aging society, coupled with the growing dependence on technology in health care, prioritizing eHealth strategies to address fear of dementia becomes crucial. This need will be further emphasized with the development of second-generation memory clinics, which target the “worried well” and as discussions on brain health extend to younger populations. It is essential for researchers, healthcare professionals, governments, and society to collaborate in developing and implementing equitable, user-friendly eHealth tools, as well as accessible manuals and video tutorials, to support older adults in managing their fear of dementia.
